# The influence of leader relational energy on employee cognitive well-being: A moderated mediation model

**DOI:** 10.3389/fpsyg.2022.940180

**Published:** 2023-02-16

**Authors:** Danping Liu, Siyuan Gui, Bingran Zhang, Haiyin Gao, Xiao Yu, Miaoxuan Wu

**Affiliations:** ^1^School of Management, Xihua University, Chengdu, China; ^2^Research Institute of International Economics and Management, Xihua University, Chengdu, China; ^3^Xihua Honor College, Xihua University, Chengdu, China; ^4^Department of Medical Administration, West China Hospital, Sichuan University, Chengdu, China; ^5^School of Health Management, Xihua University, Chengdu, China

**Keywords:** leader relational energy, work absorption, employee cognitive well-being, coworker relational energy, moderated mediation

## Abstract

To demonstrate how cognitive well-being effectively occurs, this study examines the interactive effects of relational energy on cognitive well-being. Based on conservation of resource (COR) theory, this study expands understanding of the relationship between leader relational energy and employee cognitive well-being, using 245 employees as the sample in the experiment of exploring the mediation role of work absorption. Meanwhile, the significance of co-worker relational energy is highlighted as a key boundary condition for effective leader relational energy. The results of a three-wave time study in China showed that employee work absorption played a mediating role between leader relational energy and employee cognitive well-being. In addition, co-worker relational energy moderated the relationship between leadership relational energy and work absorption. This study provides novel insights into management practice for leaders to improve employee cognitive well-being.

## 1. Introduction

Due to recent developments in the world economy and the growing complexity of work (Green and McDermott, 2010), more and more workers are experiencing negative repercussions such as burnout, interpersonal conflict, and disaffection. To cope with these issues, employees are often urged to gain greater self-awareness and develop more positive self-images (King and dela Rosa, 2019) as occupational well-being is thought to reflect one’s self-cognition level. Van Horn et al. (2004) proposed that employee occupational well-being be divided into five dimensions: affective well-being, professional well-being, cognitive well-being, social well-being, and psychosomatic well-being.

Increased cognitive demands are often viewed as inevitable outcomes of change and progress in the world of work (Korunka and Kubicek, 2017; Prem et al., 2017). In this context, cognitive well-being focuses on workers’ cognitive evaluation of psychological functions that affect mental health and self-actualization in the workplace (Ryff and Keyes, 1995; Ryan and Deci, 2001; Waterman, 2007). A high level of cognitive well-being indicates a positive perception of one’s own cognitive and mental abilities. Cognitive well-being is also a key indicator of employee potential and a driver of job satisfaction (Huang, 2014; Balzarotti et al., 2016). It even helps employees solve problems and execute complex tasks successfully. Despite its importance as a key aspect of employee occupational well-being, cognitive well-being has received little attention from scholars.

Although previous studies have examined personal factors (Robinson, 2000; Steel et al., 2008), job characteristics (Voorde et al., 2011) and external circumstances (e.g., recent life events; Diener et al., 2010) that influence employee cognitive well-being, few researchers have explored the antecedents of cognitive well-being from a social interaction perspective. Social interactions provide an energy resource that can galvanize engagement and performance (Baker et al., 2003; Cross et al., 2003; Cole et al., 2012; Owens et al., 2016), and enhance well-being (Heaphy and Dutton, 2008). Relational energy refers to psychological resourcefulness generated from interpersonal interactions that enhances one’s capacity to do work (Owens et al., 2016). Energy generated from interpersonal interactions may also improve cognitive flexibility (Baruah and Reddy, 2018).Leaders and coworkers are important purveyors of relational energy for employees in the workplace (Owens et al., 2016; Amah, 2018); however, relationships among workplace peers differ fundamentally from relationships between employees and their leaders (Chiaburu and Harrison, 2008). Contemporary work often involves group collaboration and frequent peer-to-peer interpersonal interaction (Griffin et al., 2007). But relationships between employees and leaders are largely based on economic impact and authority (Karasek et al., 1982). Salas-Vallina and Alegre (2018) argued that “specific leadership styles might contribute to employee well-being,” whereas relationships between employees are more about social reciprocity and trust (Cole et al., 2002). Indeed, it may be the quality of the social reciprocity that most significantly impacts employees (Salas-Vallina and Alegre, 2018).

Previous studies have considered relational energy in its entirety (Owens et al., 2016; Amah, 2018; Mao et al., 2022) but have not investigated the differing impacts of different forms of relational energy on employee cognitive well-being. These studies have examined relationships between leaders and employees or among coworkers (Graen and Uhl-Bien, 1995; Lian et al., 2012; Liu et al., 2013; Owens et al., 2016; Wang et al., 2018; Yang et al., 2019), but they have not examined specifically how energy from leaders or coworkers may positively influence employee cognitive well-being.

Energizers bring themselves fully to a given interaction, keeping their attention on the person or people they are involved with at the moment (Cross et al., 2003). Thus, we posit that work absorption is an important mediating mechanism in the relationship between energy and employee cognitive well-being. Work absorption refers to the extent to which employees focus on their work roles and emphasizes the intensity of focus on that role (Bloombaum and Goffman, 1962; Kahn, 1990). According to the investment principle of COR theory, individuals invest resources in order to secure more and more valuable resources (Hobfoll and Stevan, 1989). In the context of leader relational energy, COR theory holds that employees are likely to devote more psychological resources to their tasks after interacting with a high relational energy leader, thus leading to a higher state of work absorption and cognitive well-being.

**Figure 1:**
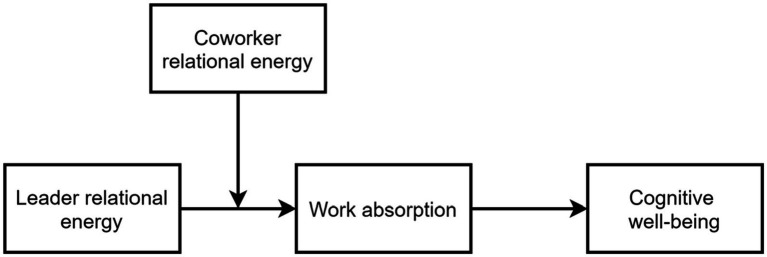
Theoretical model.

We tested these propositions in a time-lagged multisource study, and our investigation into the impacts of workplace relational energy through the lens of COR theory offers several contributions to the literature. First, our research differs from previous studies by focusing on the process of energy influence that motivates an employee’s behavior instead of taking energy as the mediating variable in facilitating work-related outcomes (Wang et al., 2018; Yang et al., 2019, 2021). In doing so, we approach the subject from a different perspective and enhance understanding of the consequences of relational energy in the workplace. Second, we incorporated coworker relational energy as well as leader relational energy and thus demonstrated the interactive effects of relational energy on workplace well-being. We also empirically answered Owens et al. (2016) call to explore coworkers as a viable source of relational energy, whereas previous research has primarily associated relational energy with leadership behavior (e.g., Graen and Uhl-Bien, 1995; Lian et al., 2012; Owens et al., 2016; Wang et al., 2018; Yang et al., 2019). Finally, we found that work absorption is an important mediating mechanism of leader/coworker relational energy on employee cognitive well-being. This finding sheds light on how relational energy influences employee cognitive well-being by suggesting that individuals derive focus from relational energy, which subsequently helps them achieve cognitive well-being. We respond to the call for greater attention to work absorption (Zou and Zuo, 2015). Figure 1 presents our proposed research model.

## 2. Theoretical background and hypotheses

### 2.1. Conservation of resources theory

COR theory suggests that individuals are motivated to retain, protect, and build resources (Hobfoll and Stevan, 1989). It consists of two competing tenets: resource conservation and resource investment. The resource conservation tenet proposes that individuals with limited resources are motivated to protect their remaining resource from further depletion.

The resource investment tenet of COR theory suggests that resources in the workplace are invested to acquire new resources when they are available (Hobfoll and Stevan, 1989). Relational energy, as a special resource, means that individuals have more resources or access to resources that can enhance their motivation to work and help them achieve their set goals (Wang et al., 2018; Yang et al., 2019; Xiao et al., 2020; Mao et al., 2022). Individuals may enhance future resources by investing energy resources they currently possess or those that are readily available from their environment. Resource surpluses, therefore, are likely to engender feelings of positive well-being (Owens et al., 2016; Shulga et al., 2022).

Rooted in the rational principle of resource maximization, COR further explains the process by which individuals actively assess their environment and are vigilant in their concerns and efforts to conserve current resources (Hobfoll, 2002; Halbesleben et al., 2014). Scholars have argued that the value of resources can change greatly depending on the setting. COR theory defines resources as things that people value (Hobfoll and Stevan, 1989), and the value of resources is determined by social or personal values, implying that relational energy, as a resource, has a lower marginal utility. Thus, co-worker relational energy may function as a key boundary condition for effective leader relational energy.

### 2.2. Leader relational energy and cognitive well-being

In a workplace setting, cognitive well-being emphasizes work-related cognitive assessments (Diener et al., 2003; Luhmann et al., 2012) and reflects the quality of employees’ cognitive efficacy (Van Horn et al., 2004; Kereste et al., 2012). Owens et al. (2016) posit that the specific energy generated from interpersonal interactions can influence employee well-being. For example, when interacting with leaders, employees receive relational energy that motivates them, enhances their capabilities, and increases their well-being (Owens et al., 2016). We propose that relational energy from leaders serves to increase employee cognitive well-being.

Social interactions can be a source of emotional energy since positive social experiences promote feelings of emotional energy (Michel et al., 2021). The concept of energy may overlap with the concept of well-being (Diener, 1984), as people high in energy may also be high in well-being (Ryan and Frederick, 1997). Previous studies have found a positive relationship between vitality and well-being (Ryan and Frederick, 1997), as people who have positive interpersonal relationships are more likely to be happy at work (Baker, 2019). Therefore, a high level of leader relational energy can evoke favorable emotional states in employees and contribute to an overall sense of well-being.

According to COR theory, Hobfoll (2011) has theorized that resources do not exist individually but travel in packs or caravans for both individuals and organizations, so the psychological resources created by such relational energy can positively influence employee cognitive well-being. That is, leader relational energy becomes individual psychological energy, making employees feel energized in their interactions with the leader and motivated to boost performance (Cole et al., 2012). Such interpersonal relationships may foster information sharing (Fredrickson, 1998) and bolster vitality and learning at work (Spreitzer et al., 2005), thereby increasing employee cognitive well-being. Thus, we hypothesize the following:

*Hypothesis 1*: Leader relational energy is positively related to cognitive well-being.

### 2.3. Leader relational energy and work absorption

Work absorption, defined as the central psychological dimension of work engagement (Rothbard, 2001; Schaufeli and Bakker, 2004), describes an employee’s psychological state and level of concentration and immersion in a given task (Dumas and Perry-Smith, 2018). We propose that the relational energy derived from a leader may increase employee work absorption.

According to COR theory, employees should allocate their resources in a way that maximizes their return and is most suited to investing in certain resources, which are frequently repurposed at work (Hobfoll, 2001). Hence COR theory (Hobfoll and Stevan, 1989; Hobfoll, 2001) has been used to explain the antecedents and consequences of work engagement (Salanova et al., 2005). Because leaders tend to control important resources in the workplace (French and Raven, 1959), energy from leaders is an important work resource and is therefore likely to affect employees’ energy levels and enthusiasm (i.e., increasing work absorption).

On the other hand, according to the investment tenet of COR theory, people must invest resources in order to acquire resources (Wheeler et al., 2013; Hobfoll et al., 2018). Employees’ psychological resources are strengthened by high leader relational energy, and they tend to reinvest such resources in their work in order to accrue additional resources. Employees influenced by leader relational energy are more likely to become attentive to their work situations and more focused (Gupta and Devalina., 2015), avoiding distractions and increasing concentration (Cole et al., 2012), which can enhance work absorption. Thus, we hypothesize the following:

*Hypothesis 2*: Leader relational energy is positively related to work absorption.

### 2.4. Work absorption and cognitive well-being

We expect work absorption to increase employee cognitive well-being for two reasons. First, according to corollary 1 of COR theory, those with greater resources are less vulnerable to resource loss and more capable of resource gain. Employees who are in a state of absorption are less prone to resource loss and more capable of organizing resources, and they are more likely to have high levels of focus, vitality, and learning (Rothbard, 2001), reach their full potential (Ilies et al., 2017), and experience true self-actualization at work, thereby improving their overall perception of work at the cognitive level of evaluation. Moreover, employees in a state of work absorption are less likely to notice external factors due to their focus on their work (Rothbard, 2001), which means they experience negative emotions less frequently. As a result, employees are more likely to enjoy cognitive well-being in states where they experience self-actualization and potential exploration at a higher frequency than when they experience negative emotions at a lower frequency.

Second, absorption as a role is intrinsically driven, and the relatively steady cognitive efficacy created by employees in the work domain, such as a greater sense of job competence and higher job satisfaction, is likely to improve in this state of focus (Deci and Ryan, 1991). As a consequence, it is clear that employees’ cognitive and action capability during the absorption stage has a significant influence on their cognitive well-being. Thus, we hypothesize the following:

*Hypothesis 3*: Work absorption is positively related to cognitive well-being.

### 2.5. The mediating role of work absorption

Building on COR theory, we argue that work absorption is a mediator in the relationship between leader relational energy and employee cognitive well-being. Due to the influence of relational energy on individual conduct throughout a contact, leader relational energy has a beneficial effect on absorption. Employees gain resources when leader relational energy is transmitted to them, and the transfer of resources conveys motivation and the ability to act (Quinn et al., 2012), which translates into work absorption behaviors, in line with COR theory. Moreover, interaction ritual theory posits that positive employee-leader contact fosters the generation of staff resources which leads to positive employee perceptions, attitudes, and behaviors (Van Vianen et al., 2018).

As a result, a leader’s relational energy increases employee work absorption. Work absorption induced by leader relational energy motivates employees to be fully engaged in their tasks and to forget about the passage of time and what is going on around them (Bloombaum and Goffman, 1962; Kahn, 1990; Van Horn et al., 2004), thereby increasing their mastery of resources. This increased degree of concentration is likely to bring out an employee’s full potential, allowing him or her to experience complete self-actualization and more positive feelings (e.g., pleasure, enjoyment). As a result, employees who are deeply engaged in their work are more likely to improve their overall perception of work at the cognitive level of evaluation. In other words, as a distinctive resource, leader relational energy may stimulate workers’ energy, encourage them to fully focus on their tasks, and generate a condition of work absorption that increases their cognitive satisfaction. Thus, we hypothesize the following:

*Hypothesis 4*: Employee work absorption mediates the relationship between leader relational energy and cognitive well-being.

### 2.6. Moderating effects of coworker relational energy

COR theory further prostulates that employees consider a variety of elements comprehensively when making both work-related (Chiu and Tsai, 2006; Halbesleben and Bowler, 2007) and interpersonal (Halbesleben and Wheeler, 2011; Unger et al., 2014) investment decisions. Schmidt and Keil (2013) argue that resource interactions can influence the value of resources. Findings from previous studies have shown that work absorption is influenced by many changeable variables, such as positive affect and social and individual resources (Christian and Slaughter, 2007; Rodríguez-Sánchez et al., 2011; van Woerkom et al., 2016). Contact with coworkers can help individuals acquire relational energy (Xiao et al., 2020) and can facilitate psychological and behavioral responses (Methot et al., 2016; Lu et al., 2017), enabling employees to engage in constructive work behaviors (Quinn et al., 2012). Thus, we posit that coworker relational energy has a substitution effect on leader relational energy since interactions with coworkers can also motivate employees to focus on their work.

This research was further extended to the interaction effect of coworker relational energy and leader relational energy on employee work absorption. The positive effect of leader relational energy on employee work absorption may become less pronounced when coworker relational energy is high because employees may not be concerned about work relationships in their job circumstances if they have access to relevant psychological resources from their colleagues (Methot et al., 2016). In this circumstance, leader relational energy loses its uniqueness and scarcity, and this reduces the weight that an employee assigns to leader relational energy. Even when leader relational energy is low, employees may rely on coworkers to maintain their concentration and focus (Christian and Slaughter, 2007). Therefore, it is suggested that high coworker relational energy may substitute for the positive effect of leader relational energy as an influence on employee work absorption.

When coworker relational energy is low, employees are unable to replenish their energy from coworkers and may tend to seek more resources from their leaders (Wright and Hobfoll, 2004). The relational energy of leadership is more convenient for employees to gain the basic resource. And then leader relational energy makes employees become more attentive and focused on the work situation (Gupta and Devalina., 2015). According to the investment tenet of COR theory, employees will put further resources into their work, seeking to avoid distraction and increase concentration (Cole et al., 2012), thereby facilitating work absorption (Cole et al., 2012). The leader’s relational energy may thus exert a stronger effect on employee work absorption. The following hypothesis is offered based on this analysis.

*Hypothesis 5*: Coworker relational energy moderates the relationship between leader relational energy and work absorption such that when coworker relational energy is low, the relationship between leader relational energy and employee work absorption is stronger.

### 2.7. Moderated mediation

According to Hypothesis 4, employee work absorption mediates the relationship between leader relational energy and cognitive well-being, while Hypothesis 5 posits that coworker relational energy moderates the relationship between leader relational energy and work absorption. Taken together, we propose a moderated mediation model.

Employees can obtain energy replenishment from coworkers when coworker relational energy is high, resulting in less need for psychological resources to be replenished by leaders and making it difficult for leader relational energy to motivate employees to produce a state of work absorption and thereby improve cognitive well-being. However, when coworker relational energy is low, leader relational energy is more crucial based on the basic need for energy replenishment. Thus, employees are influenced by leader relational energy to a greater extent and are able to attain a positive state of work absorption, Intermediary intensity increases, which in turn improves the cognitive level indicators and produces cognitive well-being. Thus, we hypothesize the following:

*Hypothesis 6*: Coworker relational energy negatively moderates the mediating role of work absorption between leader relational energy and cognitive well-being such that greater coworker relational energy mitigates the mediating role of work absorption between leader relational energy and cognitive well-being.

## 3. Materials and methods

### 3.1. Participants and procedures

We tested our theoretical model by conducting an online survey on social media platforms in China. Using a snowball sampling method (Biernacki and Waldorf, 1981), we sent a survey link to MBA students with full time jobs and encouraged them to share the link with others who were interested in the program and demonstrated suitable interest in the questionnaire. The majority of participants came from the financial, technology and education sectors. Participants were given 15 Chinese Yuan as appreciation for voluntarily taking part in the study.

To reduce common method variance bias, we conducted a three-time lagged survey to examine our hypotheses. At Time 1, participants responded to questions regarding demographic variables, leader relational energy and coworker relational energy. At Time 2, 1 month later, participants rated their work absorption during the month. At Time 3, we assessed employee cognitive well-being. We collected 289 questionnaires at Time 1, 274 questionnaires at Time 2 (for a response rate of 94.81%), and 253 questionnaires at Time 3 (for a response rate of 92.337%). After excluding surveys that did not pass the attention check, our final study sample was 245. Among the 245 employees, 28.6% were male, and 65.7% had a bachelor’s degree. Average age was 31.840 and average tenure was 6.703 years.

### 3.2. Measure

The scales used in this research were derived from previous studies. A back-translation process was adopted to obtain the final scales for the study (Brislin, 1986). All variables were evaluated using a five-point Likert scale from 1 (strongly disagree) to 5 (strongly agree). See the Appendix for a complete listing.

#### 3.2.1. Relational energy

Relational energy was measured with Owens et al. (2016) five-item scale. We divided relational energy into two dimensions: leader relational energy and coworker relational energy. The sample items from the scale were “I feel invigorated when I interact with my leader” and “I feel increased vitality when I interact with my coworkers.” Cronbach’s α were 0.904 and 0.902, respectively.

#### 3.2.2. Work absorption

Work absorption was assessed by employees using an established five-item scale from Rothbard (2001). A sample item is “when I was working, I was totally absorbed by it.” Cronbach’s α was 0.715.

#### 3.2.3. Cognitive well-being

Cognitive well-being was measured by a five-item scale developed by Huang (2014). A sample item is “I can concentrate easily.” Cronbach’s α was 0.849.

#### 3.2.4. Control variables

Based on prior studies (Yang et al., 2018; Fan et al., 2021), we controlled for participants’ gender, age, education level, and years of work experience.^1^

### 3.3. Analytic strategy

Regression analysis was conducted to examine our theoretical model with SPSS 26.0 and Mplus 7.0. First, we used the Herman one factor analysis to test for common method bias. Second, we conducted CFA testing to measure the discriminant validity of the model. Third, we did correlation analysis using SPSS 26.0, and then we used the indirect effect of work absorption with Mplus 7.0 by employing a bias-corrected bootstrap confidence interval. Finally, the interaction of leader relational energy and coworker relational energy was derived to test the moderating effect.

## 4. Results

### 4.1. Common method deviation test

Although we collected questionnaires at three different points in time, the questionnaires were all completed by employees, and so the study results could be subject to common method variance bias. In order to control for common method variance bias, we used Herman one factor analysis. The variance explained by the analyzed main factors was 33.976%, which is less than 40% of the total explained variance. We then combined all factors into one item and formed a five-factor model. After adding the common latent factor, the fit index of the five-factor model was χ2 = 263.54, *df* = 126, CFI = 0.92, TLI = 0.89, RMSEA = 0.07, SRMR = 0.05. Compared to the four-factor model (χ2 = 191.58, *df* = 142, CFI = 0.97, TLI = 0.96, RMSEA = 0.04, SRMR = 0.05), the fit index is worse. Thus, we argue that common method bias does not constitute a serious problem in the study.

### 4.2. Confirmatory factor analysis

The effectiveness of the differentiation between variables was tested using Mplus 7.0. Compared with other models (see Table 1), the four-factor model had the best fit index (=191.58, df = 142, CFI = 0.97, TLI = 0.96, SRMR = 0.05, RMSER = 0.04), suggesting a clear distinction between the four variables.

**Table 1:** Confirmatory factor analysis.

Model	χ^2^	*df*	χ^2^/ *df*	SRMR	RMSEA	CFI	TLI
Four-factor model	191.58	142	1.35	0.05	0.04	0.97	0.96
Three-factor model	659.96	149	4.43	0.10	0.12	0.69	0.64
Two-factor model	778.70	151	5.16	0.12	0.13	0.62	0.57
One-factor model	1076.20	152	7.08	0.14	0.16	0.43	0.36

*N* = 245. Four-factor model = leader relationship energy, coworker relationship energy, work absorption, cognitive well-being. Three-factor model = leader relationship energy + coworker relationship energy, work absorption, cognitive well-being. Two-factor model = leader relationship energy + coworker relationship energy, work absorption + cognitive well-being. One-factor model = leader relationship energy + coworker relationship energy + work absorption + cognitive well-being.

### 4.3. Preliminary analyses

We report the AVE and CR results in Table 2 below. According to Fornell and Larcker (1981), composite reliability (CR) must be more than 0.6, and AVE must be more than 0.5. Our results indicate that the variables in our study have acceptable reliability and validity. Mean, standard deviation, and correlation coefficient are shown below in Table 3. Leader relational energy was significantly positively correlated with work absorption (*r* = 0.28, *p* < 0.01) and cognitive well-being (*r* = 0.25, *p* < 0.01); Work absorption was significantly positively correlated with cognitive well-being (*r* = 0.26, *p* < 0.01).

**Table 2:** Results of reliability and convergence validity of each variable.

Variables	Cronbach’s α	CR	AVE
Leader relational energy	0.90	0.91	0.66
Coworker relational energy	0.90	0.90	0.65
Work absorption	0.72	0.89	0.67
Cognitive well-being	0.85	0.85	0.54

*N* = 245.

**Table 3:** Descriptive statistics and correlations of current study.

Variable	M	SD	1	2	3	4	5	6	7	8
1. Gender	1.71	0.45	1							
2. Age	31.84	6.77	−0.11	1						
3. Education	4.37	1.01	−0.03	0.11	1					
4. Work years	6.70	7.20	−0.12	0.83**	0.14*	1				
5. Leader relationship energy (T1)	3.17	0.77	−0.09	0.12	−0.08	0.09	1			
6. Coworker relationship energy (T1)	3.47	0.62	0.00	−0.04	−0.15*	−0.02	0.49**	1		
7. Work absorption (T2)	3.22	0.69	−0.08	0.04	−0.13*	0.08	0.28**	0.27**	1	
8. Cognitive well-being (T3)	3.71	0.52	−0.06	0.18**	−0.02	0.18**	0.25**	0.29**	0.26**	1

*N* = 245. *p < 0.05; **p < 0.01.

### 4.4. Tests of hypotheses

**Figure 2:**
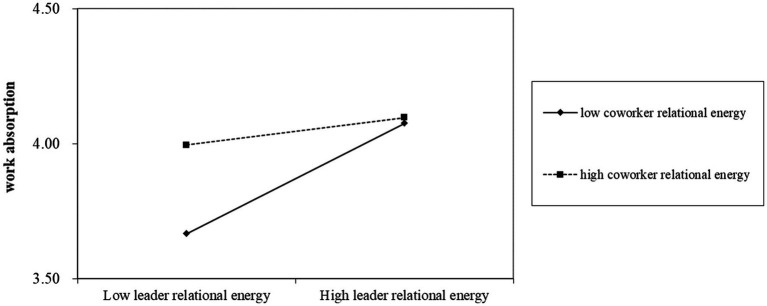
The interactive effect of leader relational energy and coworker relational energy on work absorption.

Table 4 shows the unstandardized coefficient result of regression analysis. Table 5 displays the indirect effects and conditional indirect effects. Figure 2 displays the moderating effect.

**Table 4:** Results of regression analysis.

Predictors	Work absorption		Cognitive well-being	
Model	M1	M2	M3	M4
Intercept	3.30***	3.96***	3.10***	2.61***
Gender	−0.09	−0.08	−0.03	−0.01
Age	−0.01	−0.01	0.01	0.01
Education	−0.08	−0.08	−0.01	0.00
Work years	0.02	0.02	0.01	0.01
Leader relationship energy	0.24***	0.17*	0.15***	0.12**
Work absorption				0.15**
Coworker relationship energy		0.14		
Leader relationship energy × Coworker relationship energy		−0.16*		
*R* ^2^	0.11**	0.15*	0.09*	0.13**

*N* = 245. *p < 0.05. **p < 0.01. ***p < 0.001.

**Table 5:** Summary of indirect effects and conditional indirect effects.

Paths and effects	Estimates	SE	95% confidence intervals
Leader relationship energy → Work absorption → Cognitive well-being			
Indirect effects	0.04*	0.02	[0.001, 0.08]
Moderated mediation			
High coworker relationship energy	0.06	0.06	[−0.04, 0.18]
Low coworker relationship energy	0.16*	0.07	[0.05, 0.30]
Indirect difference	−0.1	0.09	[−0.28, 0.07]

*N* = 245. *p < 0.05. **p < 0.01. ***p < 0.001.

Regarding Hypothesis 1 that leader relational energy is positively associated with employee cognitive well-being, Table 4 shows that leader relational energy is positively associated with cognitive well-being (B = 0.15, *p* < 0.001), supporting Hypothesis 1. The second hypothesis pertains to the relationship between leader relational energy and work absorption. According to Table 4, leader relational energy is positively related to work absorption (B = 0.24, *p* < 0.001) in support of Hypothesis 2. Hypothesis 3 proposes that work absorption is positively related to cognitive well-being. Based on Table 4, work absorption positively predicted employee cognitive well-being (B = 0.15, *p* < 0.01). Thus, these results support Hypothesis 3. The fourth hypothesis tested the mediating role of work absorption. As shown in Table 5, work absorption mediated the positive relationship between leader relational energy and employee cognitive well-being (indirect effect =0.04, 95% CI = 0.001, 0.08), supporting Hypothesis 4. Hypothesis 5 predicted that coworker relational energy moderates the relationship between leader relational energy and work absorption. As seen in Table 4 and Figure 2, the moderating effect on the link between leader relational energy and work absorption was negatively significant (B = -0.16, p < 0.01), supporting Hypothesis 5. Hypothesis 6 examined the conditional indirect effect of work absorption. As shown in Table 5, the indirect effect of leader relational energy on employee cognitive well-being *via* work absorption was not significant at higher levels of coworker relational energy (indirect effect =0.06, 95% CI = −0.04, 0.18) but was significant at lower levels of coworker relational energy (indirect effect =0.16, 95% CI = 0.05, 0.30). However, the difference was not significant (indirect effect = −0.10, 95% CI = −0.28, 0.07), and therefore Hypothesis 6 is not supported by the results.

## 5. Discussion

Based on COR theory, we aimed to test the interaction effect of two fundamental relational energies on employee cognitive well-being through a questionnaire survey at three points in time. Our results indicate that leader relational energy generally is positively associated with employee cognitive well-being *via* work absorption, reflecting the importance of relational energy and echoing the research outcomes of many other scholars (Yang et al., 2019; Xiao et al., 2020). Coworker relational energy moderates the relationship between leader relational energy and employee work absorption such that the relationship is weaker when coworker relational energy is high. This is consistent with Winkel et al. (2011)‘s study and suggests that the value of resources changes depending upon the setting.

When coworker relational energy is low, coworker relational energy negatively moderates the mediating role of work absorption between leader relational energy and cognitive well-being such that the lower the coworker relational energy, the larger the mediating role of work absorption between leader relational energy and cognitive well-being.

The moderated mediation model, however, fails the test when coworker relational energy is large. When coworker relationships are strong, leader relationships are less significant. At this point, a leader’s connection energy could negatively impact cognitive health. The availability of energy resources is limited, according to ten Brummelhuis and Bakker (2012), and employees’ capacity to absorb work-related energy from coworker relationships when it is strong is severely constrained. The relationship between mood and performance was found to be more susceptible to influence from supervisors than interactions with coworkers (McCarthy et al., 2016). When interacting with those who have relatively more power, employees are prone to hide their negative feelings, which is obviously counterproductive to developing a deeper sense of self.

### 5.1. Theoretical implications

This study offers several theoretical contributions. First, it identifies how leader relational energy contributes to employee cognitive well-being (Diener, 1984; Lucas et al., 1996; Schimmack, 2008; Busseri and Sadava, 2011). Cognitive well-being, an important indicator of employee potential, can explain from a motivational perspective the mechanism of happiness at work (Huang, 2014). Empirical research has shown that cognitive well-being is highly correlated with comprehensive assessments of satisfaction in important life domains (Schimmack et al., 2008). As energy is a vital life resource, it may impact cognitive evaluation. However, the mechanism underlying the relationship between relational energy and cognitive well-being is not well understood. Therefore, based on COR theory, this research combines positive psychology and human resource management and investigates how relational energy influences employee cognitive well-being through the lens of interpersonal interaction. Our results indicate that leader relational energy is positively related to cognitive well-being, a finding that reflects the importance of relational energy and echoes the research outcomes of many other scholars (Yang et al., 2019; Xiao et al., 2020). However, unlike previous studies that view relational energy as an intermediary variable and positive outcomes as merely a result of what one invests in such interactions rather than what one receives from them (Brown et al., 2003), our findings strengthen understanding of the consequences of relational energy. This research responds to Salas-Vallina and Alegre (2018) appeal that “HRM research needs to prioritize increased employee well-being” as employees perform better in the long run if organizations prioritize their well-being. It also enriches the canon of research on human resource management and well-being.

Second, this study addresses an important relational energy question: how do different sources of relational energy influence employee behavior? Most prior research has focused only on dyadic relationships between leaders and employees or individuals and colleagues (Graen and Uhl-Bien, 1995; Lian et al., 2012; Owens et al., 2016; Wang et al., 2018; Yang et al., 2019), without distinguishing between the sources of energy. In contrast, we have considered both fundamental sources of work-related relational energy, leader relational energy and coworker relational energy, and examined the interaction impact on employee cognitive well-being. Thus, we have refined the empirical understanding of relational energy by testing the energy interactions of leaders and coworkers in response to a research gap noted by Owens et al. (2016). The results of this study show that employees can boost work absorption by assimilating relational energy from either leaders or colleagues in their work environment. Furthermore, when coworker relational energy is low, obtaining energy resources from the leader becomes more important. In this situation, low coworker relational energy increases an employee’s cognitive well-being. In the case of high coworker relational energy, the relational energy is less important and its scarcity decreases, thus reducing the effect of relational energy. This finding implies that the effect of relational energy can be influenced by organizational circumstances. This is consistent with Winkel et al. (2011)‘s study and suggests that the value of resources changes depending upon the setting. The identification of this important boundary condition helps clarify constraints on the effect of leader relational energy on cognitive well-being and extends the research in this area.

Finally, this study examines the mediating role of work absorption from a COR theory perspective and responds to the call for greater attention to work absorption (Zou and Zuo, 2015), given that work absorption is a key aspect of work engagement (Schaufeli and Bakker, 2004; Bakker, 2011). These results indicate that work absorption plays a mediating role in the relationship between leader relational energy and employee cognitive well-being. According to COR theory (Hobfoll, 2001), when employees acquire relational energy from a leader, they can use the resources to generate a state of focus on work through self-investment, which in turn leads to increased cognitive well-being. This study, thus, unlocks the black box of the influence of leadership relational energy on cognitive well-being and provides a new perspective for future research. In doing so, we respond empirically to Baker (2019) call to investigate the transfer mechanisms of relational energy.

### 5.2. Practical implications

This study has several implications for organizational managers. First, workplace well-being is the glue that holds organizations together and enables firms to retain and reward high-quality workers (Fisher, 2010). Cognitive well-being permeates individuals’ work and family lives as an important component of well-being (Dierendonck, 2004; Van Horn et al., 2004). Therefore, companies should seek to better understand its importance and improve their employees’ cognitive well-being. Companies can implement job-related cognitive skills training and concentration training programs to boost employees’ cognitive effectiveness. In addition, managers should ensure that employees have sufficient resources to appreciate the meaning and value of their work.

Second, these findings suggest that leader relational energy can increase employee cognitive well-being. Therefore, organizations should look for leader candidates with high relational energy. Leaders themselves should strive to maintain open, positive communication and interaction channels with employees, and managers should take steps to create a harmonious and united atmosphere that is conducive to good relationships with subordinates.

Finally, because employee relational energy can be absorbed from colleagues, managers should endeavor to support good employee peer-to-peer relations. Prior research confirms that open communication is one key to maintaining such relationships (Miles et al., 1996). In order to sustain a harmonious working environment, managers can improve team cohesion through quality and team-building activities. When coworker relational energy is low, the effect of leader relational energy on employee cognitive well-being through work absorption increases, and when coworker relational energy is high, the influence is less. This suggests that managers should employ different managerial strategies for employees with high vs. low coworker relational energy. For employees with high coworker relational energy, managers should delegate authority appropriately, enhance communication between employees through empowerment, and provide employees with adequate work resources, such as supervisory support. For employees with low coworker relationship energy, leaders can encourage peer-to-peer interactions, support employee self-improvement, and convey a sense of trust to improve relational energy.

### 5.3. Limitations and future research directions

While this study focuses on the link between leader relational energy and employee cognitive well-being, future research could examine connections between energy and other dimensions of well-being, such as emotional well-being, occupational well-being, and social well-being. Moreover, this study did not examine other antecedents of employee cognitive well-being, such as individual characteristics (e.g., gender, age, education level, etc.) or characteristics of the organization (e.g., nature of ownership, industry or sector, stage of development, etc.). We encourage researchers to further examine the variability of cognitive well-being among different groups and organizations in the future.

Second, this study examines the positive effects of relational energy from the perspective of the energy receiver. However, conveying energy to others may have negative effects on energy providers. Owens et al. (2016) argued that individuals with high energy transfer capacity may deplete their own energy and reduce their ability to self-regulate. Future research may explore the potential negative effect of relational energy from the perspective of the energy provider. In addition, as most work environments require individuals to be integrated into teams and departments (Cole et al., 2012), exploring energy expressions at the interaction and collective levels may better explain organizational phenomena, and research on relational energy and collective energy calls for greater attention (Zhu et al., 2017; Baker, 2019). Future research can explore team energy and the dual effects of relational energy in organizations.

Finally, although this study used a three-time lagged design, which reduces common method variance bias to some extent, this research relied on employees’ self-rated data collection. Therefore, common method variance bias cannot be completely ruled out. We encourage scholars to conduct multi-source or longitudinal investigations in the future to examine the relationship between leader relational energy and employee cognitive well-being.

## 6. Conclusion

This study enriches the body of knowledge about the relationship between leader relational energy and employee cognitive well-being by probing the mediating role of work absorption. Moreover, this research highlights the importance of coworker relational energy as a key boundary condition to the effectiveness of leader relational energy. Individuals with low coworker relational energy are more likely to accrue work absorption from a leader’s relational energy, which in turn increases employee cognitive well-being. Our findings offer ways for managers to successfully improve their employees’ cognitive well-being.

## Data availability statement

The original contributions presented in the study are included in the article/supplementary material, further inquiries can be directed to the corresponding author.

## Author contributions

DL reviewed the literature, proposed the research model, and designed the study. SG conducted the literature search, drafted the manuscript and edited it. BZ conducted the data analysis. HG put forward many constructive suggestions for revision of the manuscript. XY participated in revision of the manuscript. MW revised the manuscript and rechecked the relevant data of the manuscript. All authors discussed, finalized, and approved the manuscript for publication.

## Funding

This work was supported by the Innovation Fund of Research Institute of International Economics and Management, Xihua University (Grant No. 20210014).

## Conflict of interest

The authors declare that the research was conducted in the absence of any commercial or financial relationships that could be construed as a potential conflict of interest.

## Publisher’s note

All claims expressed in this article are solely those of the authors and do not necessarily represent those of their affiliated organizations, or those of the publisher, the editors and the reviewers. Any product that may be evaluated in this article, or claim that may be made by its manufacturer, is not guaranteed or endorsed by the publisher.

## Footnotes

^1^The results showed that the inclusion of control variables did not change the results of the study. Therefore, the data reported in this paper are the results after the addition of control variables.

## Appendix

Survey items.

Respondents rated these items on a 5-point Likert scale from “strongly disagree” to “strongly agree.”

Leader relational energy:

1. I feel invigorated when I interact with my leader.

2. After interacting with my leader I feel more energy to do my work.

3. I feel increased vitality when I interact with my leader.

4. I would go to my leader when I need to be “pepped up.”

5. After an exchange with my leader I feel more stamina to do my work.

Coworker relational energy:

1. I feel invigorated when I interact with my coworker.

2. After interacting with my coworker I feel more energy to do my work.

3. I feel increased vitality when I interact with my coworker.

4. I would go to my coworker when I need to be “pepped up.”

5. After an exchange with my coworker I feel more stamina to do my work.

Work absorption:

1. When I am working, I often lose track of time.

2. I often get carried away by what I am working on.

3. When I am working, I am completely engrossed by my work.

4. When I am working, I am totally absorbed by it.

5. Nothing can distract me when I am working.

Cognitive well-being:

1. When I am working, I can concentrate easily.

2. When I am working, I feel that I can think clearly.

3. When I am working, I feel easy to concentrate on thinking.

4. When I am working, I feel comfortable solving complex problems when thinking.

5. When I am working, I have confidence in my ability to think about complex problems at work.
